# Unraveling the Predictive Value of the Novel Global Immune-Nutrition-Inflammation Index (GINI) on Survival Outcomes in Patients with Grade 4 Adult-Type Diffuse Gliomas

**DOI:** 10.3390/curroncol31090372

**Published:** 2024-08-28

**Authors:** Asim Armagan Aydin, Ramazan Oguz Yuceer

**Affiliations:** 1 Department of Clinical Oncology, Antalya Education and Research Hospital, Health Science University, 07100 Antalya, Turkey; 2 Department of Pathology, Cumhuriyet University School of Medicine, 58140 Sivas, Turkey; r.yuceer66@hotmail.com

**Keywords:** glioblastoma, global immune-nutrition-inflammation index, survival, central nervous system, brain tumor, adult-type diffuse glioma, astrocytoma, biomarker

## Abstract

Background: This investigation evaluated the predictive and prognostic efficacy of the newly developed global immune-nutrition-inflammation index (GINI) in patients with grade 4 adult-type diffuse gliomas, comparing it with other established indices such as the systemic immune-inflammation index (SII), systemic inflammation response index (SIRI), and pan-immune-inflammation value (PIV). Method: A retrospective cohort included 198 patients diagnosed with isocitrate dehydrogenase (IDH)-mutant gr4 (grade 4) astrocytoma and IDH-wt (wilde-type) glioblastoma (GBM) gr4 treated with surgical resection, radiotherapy, and temozolomide. Patients were stratified into two groups based on their GINI values: low GINI (<5815) and high GINI (≥5815). The primary endpoint was overall survival (OS). Results: High GINI was significantly associated with older age, poor performance status, multifocal tumors, and higher SII, SIRI, and PIV values (*p* < 0.005). The GINI demonstrated strong correlations with SII (r = 0.694), SIRI (r = 0.516), and PIV (r = 0.657) (*p* < 0.001). Patients with high GINI exhibited poorer OS (5.0 vs. 17.0 months) and PFS (5.0 vs. 13.0 months) in comparison to those with low GINI. Kaplan–Meier survival analysis revealed significantly prolonged OS and PFS among patients with low GINI (*p* < 0.001). Multivariate analysis identified high GINI as an independent negative risk factor for both PFS and OS. Conclusions: GINI is a robust predictor of clinical outcomes in IDH-mutant gr4 astrocytoma and IDH-wt GBM gr4, highlighting the crucial impact of nutrition and cancer cachexia. It shows superior prognostic value relative to the SII, SIRI, and PIV.

## 1. Introduction

Grade 4 adult-type diffuse gliomas, which include both isocitrate dehydrogenase (IDH) mutant gr (grade) 4 astrocytoma and IDH-wilde-type (wt) glioblastoma (GBM) gr4 according to to the fifth edition of the World Health Organization (WHO) Classification of Tumors of the Central Nervous System (CNS) criteria, as published in 2021 [1], one of the most frequent primary brain tumors, typically occur in individuals of advanced age, with an average age of 64 years [2]. They exhibit a higher incidence among males and constitute nearly half of all malignant tumors affecting the central nervous system [3,4]. The symptoms of aggressive and rapidly growing tumors generally depend on tumor localization and size [5]. The associated neurological manifestations contribute to high morbidity and mortality rates, with an overall survival of approximately 9–15 months [2,4,5]. The standard treatment consists of maximal safe resection followed by radiotherapy plus concomitant and adjuvant temozolomide [6]. Progression is almost inevitable despite treatment, and survival remains limited even with subsequent therapies [7]. Therefore, in recent years, similar to many other cancers, there has been a focus on genetic mutational changes and the development of drugs targeting these alterations. The study by Mellinghoff et al. [8], which demonstrated the efficacy of ivosidenib in IDH mutant gliomas, is the most striking example. Despite the rapid advancement of clinical trials, including targeted therapies and immunotherapies [9,10], uncertainty regarding reliable and validated biomarkers capable of predicting treatment response and clinical outcomes in the majority of patients highlights a promising area for future oncology research.

Chronic inflammation contributes to the development and progression of cancer through mechanisms such as DNA damage and mutational changes; inhibition of apoptosis by cytokines and growth factors activated by inflammation; activation of angiogenesis via mediators such as prostaglandins; inhibition of immune recognition; destruction of cancer cells due to altered immune responses; and support of tumor growth and spread through microenvironmental changes [11,12]. Owing to their critical roles in the inflammatory response, platelets, neutrophils, monocytes, and lymphocytes, which exhibit distinct characteristics that influence the immune system, constitute the primary components of peripheral blood elements in this process [13]. Moreover, the cytokines and chemokines secreted by these cells, as well as the acute-phase proteins produced by various cells through diverse mechanisms (e.g., C-reactive protein, fibrinogen, and albumin), play significant roles in mediating the body’s inflammatory response [14]. Additionally, these factors have been correlated with an elevated risk of tumor progression and adverse prognostic outcomes in GBM [15].

The results of various studies and meta-analyses, wherein combinations of these markers are readily measurable through peripheral blood and biochemical analyses, are derived from their fluctuating values, including the neutrophil-to-lymphocyte ratio (NLR), platelet-to-lymphocyte ratio (PLR), monocyte-to-lymphocyte ratio (MLR), albumin-to-globulin ratio (AGR), systemic immune-inflammation index (SII), systemic inflammation response index (SIRI), and pan-immune-inflammation value (PIV), and consistently indicate that these indices reflecting inflammation exhibit significant prognostic and predictive capabilities in the management of GBM [16,17,18,19,20,21,22,23,24,25]. The findings derived from these investigations corroborate the concept that scores computed using triple-marker formulations such as the SII, SIRI, and PIV offer more robust prognostic and predictive insights into glioblastoma survival and clinical outcomes than indices generated from binary marker combinations, such as NLR, PLR, and MLR. Albumin is predominantly synthesized in the liver and comprises the majority of the body’s total protein content, functions as a negative acute-phase reactant, and is closely associated with nutritional status. Moreover, it has been extensively validated as a prognostic biomarker in oncological studies [26,27]. In clinical settings, the C-reactive protein (CRP)-to-albumin ratio (CAR) has been investigated as a prognostic marker for diverse diseases, including cancer. Elevated CAR has been linked to unfavorable outcomes and heightened mortality in cancer patients, which is likely attributable to the synergistic effects of chronic inflammation and nutritional deficiencies on treatment efficacy and disease advancement [28].

Expanding on this hypothesis and seeking to identify novel and more powerful indices constructed from combinations of additional markers representing the immune response in inflammation, we designed a study to examine the influence of the global immune-nutrition-inflammation index (GINI), originally introduced by Topkan et al. [29], on survival and clinical outcomes among individuals diagnosed with IDH-mutant gr4 astrocytoma and IDH-wt GBM gr4. In a study conducted by Topkan et al., the GINI was found to be a marker for predicting survival in patients with non-small-cell lung cancer (NSCLC) [29].

In the current study, with the primary endpoint delineated as overall survival, we further explored the associations between the GINI and other well-established prognostic indices (such as the SII, SIRI, and PIV), along with clinicopathological characteristics. Moreover, a detailed comparative power analysis was performed between the GINI and the others. Understanding the role of the GINI and other inflammatory markers in IDH-mutant gr4 astrocytoma and IDH-wt GBM gr4 could enhance risk stratification and treatment selection and ultimately improve the care and prognosis of patients with this aggressive form of brain tumor. To the best of our knowledge, this study is the first to examine the prognostic and predictive effects of the GINI in patients diagnosed with gr4 adult-type diffuse glioma.

## 2. Materials and Methods

This retrospective study inlcluded patients who received follow-up care at the Oncology Department of Health Science University Antalya Education and Research Hospital (HSUAERH) between January 2013 and December 2022. Patients were diagnosed with IDH-mutant gr4 astrocytoma and IDH-wt GBM gr4, underwent surgical resection, and subsequently received radiotherapy in combination with temozolomide as part of the standard treatment protocol, followed by adjuvant temozolomide.

**Figure 1:**
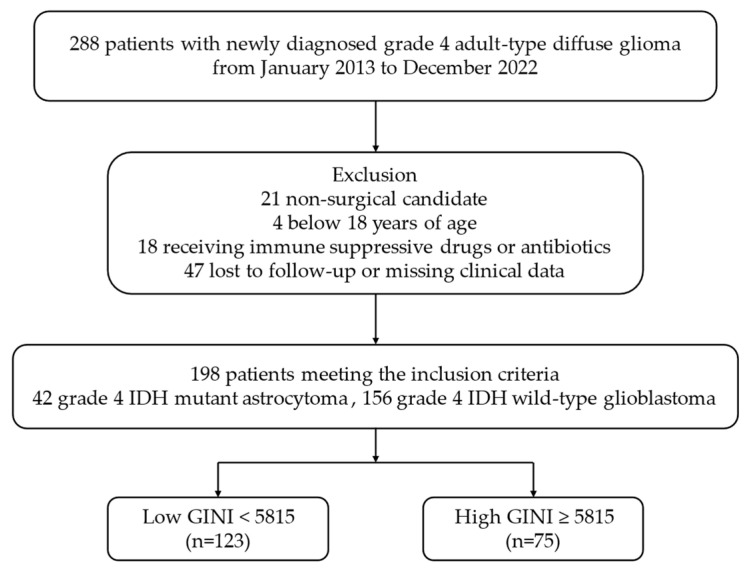
Flowchart of the study according to CONSORT diagram. Abbreviations: IDH, isocitrate dehydrogenase; GINI, global immune-nutrition-inflammation index; CONSORT, consolidated standards of reporting trials.

Data were collected from 288 patients with pathologically confirmed IDH-mutant gr4 astrocytoma and IDH-wt GBM gr4 according to the fifth edition of the WHO Classification of Tumors of the CNS criteria, as published in 2021 [1]. All the patients underwent preoperative brain magnetic resonance imaging (MRI). Comprehensive biochemical tests, including complete blood count, uric acid, lactate dehydrogenase (LDH), albumin, and CRP, were conducted concurrently within 7–10 days prior to the surgical intervention. Patients diagnosed with chronic immune or inflammatory diseases, proven active acute infections, a history of medications that could affect immune and inflammatory responses (such as steroids or antibiotics) within the last month, or those who had undergone blood transfusions in the last three months were excluded from the study. The final analysis included 198 patients for whom complete clinical and laboratory data were available. The study design and criteria are summarized in the flowchart shown in Figure 1.

After reviewing the clinical, laboratory, and radiological records of the patients, the following data were collected: age, sex, Eastern Cooperative Oncology Group Performance Status (ECOG PS), body mass index (BMI), comorbidity, smoking status, primary or secondary development of the disease, presence of neurological deficits or epileptic seizures at diagnosis, tumor lateralization, tumor-originating lobe, tumor focality, primary tumor size, type of surgical procedure, presence of alpha thalassemia/mental retardation syndrome X-linked (ATRX) loss, IDH mutation status, adjuvant radiotherapy, adjuvant chemotherapy, the presence of neutropenia development during adjuvant therapy, response after adjuvant therapy, progression development during follow-up, treatment options available at progression, and overall survival duration.

The GINI was calculated using the formula proposed by Topkan et al. [29]: GINI = (neutrophils × platelets × monocytes × C-reactive protein) ÷ (lymphocytes × albumin). Additionally, the SII was calculated as NLR × platelets [19,24], the SIRI as NLR × monocytes [22], and the PIV as NLR × platelets × monocytes [25]. Fundamentally, the GINI is formulated as a composite metric incorporating PIV, an immune-inflammatory marker, and CAR, which serves as an indicator of the nutritional status (GINI = PIV × CAR).

In the schematic representation concerning the formulation of the GINI, the variables N, P, M, CRP, L, and A denote the respective values of neutrophils (10^3^/mm^3^), platelets (10^3^/mm^3^), monocytes (10^3^/mm^3^), C-reactive protein (mg/L), lymphocytes (10^3^/mm^3^), and albumin (g/L).

GINI = N × P × M × CRP/L × A

Following the initial clinical assessment, all patients received the standard treatment protocol according to the National Comprehensive Cancer Network (NCCN) recommendations, which included radiotherapy concurrent with temozolomid followed by adjuvant temozolomide. Following the completion of radiotherapy, response assessment was conducted using serial MRI scans. The hematological side effects of adjuvant therapy were also recorded. Clinical responses were assessed and categorized as complete response (CR), partial response (PR), stable disease (SD), or progressive disease (PD), according to the revised Response Evaluation Criteria in Solid Tumors (RECIST) guidelines (version 1.1). Progression-free survival (PFS) was defined as the time elapsed from the date of pathological diagnosis to the date of progression, death, or the last visit. Overall survival (OS) was calculated as the time elapsed from the date of pathological diagnosis to the date of death from any cause or last visit.

Ethical considerations were adhered to throughout this study, which was conducted in compliance with the Helsinki Declaration of 1964, as revised in 2013. The study protocol was thoroughly reviewed and approved by the Institutional Review Board of the HSUAERH (Approval Number: 2023-314). Given the retrospective design of the study, patient consent was not mandated. Nonetheless, to safeguard patient confidentiality, the data were anonymized.

### Statistical Analysis

Statistical analyses were performed using the Statistical Package for Social Sciences (SPSS) version 27 software for Windows (IBM SPSS Inc., Chicago, IL, USA). The normal distribution suitability of continuous data was evaluated using the Kolmogorov–Smirnov and Shapiro–Wilk tests. Numerical variables conforming to a normal distribution are expressed as mean ± standard deviation, whereas those deviating from normality are presented as median (min–max). The predictive accuracy of the GINI, SII, SIRI, and PIV for mortality was evaluated using Receiver Operating Characteristic (ROC) curve analysis. The optimal cutoff values for the GINI, SII, SIRI, and PIV ratios were determined using the Youden Index method within the ROC curve analysis. Patients were stratified into two cohorts based on a GINI value threshold of 5815 or above and those below this threshold. Subsequently, these groups were compared in terms of PFS and OS rates. The relationship between the GINI and clinicopathological characteristics, as well as biochemical data, was assessed using the chi-square test, Fisher’s exact test, Mann–Whitney U test, and Kruskal–Wallis test. Spearman’s correlation coefficient was used to compare frequency distributions across the tested groups. PFS and OS were estimated using the Kaplan–Meier method and compared using the log-rank test. Variables significantly associated with survival in univariate analysis were further analyzed using multivariate Cox regression models. Statistical significance was defined as *p* < 0.05 for all analyses.

## 3. Results

In this retrospectively designed study, out of the 288 patients diagnosed with IDH-mutant gr4 astrocytoma and IDH-wt GBM gr4 who were initially screened, 21 patients ineligible for surgery, 4 patients under 18 years of age, 18 patients using chronic immunosuppressive drugs or antibiotics, and 47 patients with incomplete clinical and laboratory data during follow-up were excluded from the study. A total of 198 patients who met all criteria were included in the final analysis (Figure 1).

The median age of the cohort was 60, ranging from 25 to 86 years. The male population comprised 57.6% of the patients. Comorbid conditions were identified in 38.9% of the patients, and 33.8% had a documented history of smoking. Among the comorbid conditions, cardiovascular diseases were the most common, affecting 31 patients, followed by diabetes in 19 patients and chronic obstructive pulmonary disease (COPD) in 14 patients. Left hemispheric involvement was observed in 53.5% of the cases, with the parietal lobe being the most common tumor site (38.4%) and the occipital lobe the least commonly affected (10.6%). A unifocal tumor presentation was noted in 77.3% of the patients. The proportions of patients who underwent subtotal and gross total surgical resection were comparable. Standard radiotherapy was completed by 178 patients (89.9%) and adjuvant temozolomide therapy was administered to 159 patients (80.3%). IDH mutations were detected in 42 patients (21.2%) and ATRX loss was identified in 64 patients (32.3%). Table 1 provides a detailed summary of the sociodemographic and clinicopathological characteristics of the patients categorized according to the GINI cutoff value.

### 3.1. Cutoff Values of the Laboratory Parameters

**Figure 2:**
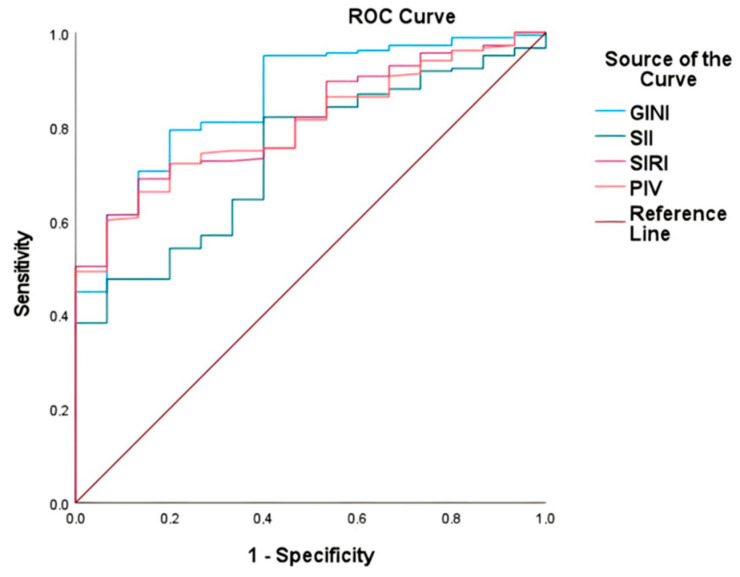
Comparison of the capability of GINI, SII, SIRI, and PIV to predict mortality in gr4 adult-type diffuse glioma using ROC curve analysis.

The GINI, SII, SIRI, and PIV indices were assessed for their predictive efficacy with respect to mortality using ROC curve analysis (Table 2). The GINI exhibited the highest area under the ROC curve (AUC) at 0.86 (95% CI: 0.78–0.95), followed by the SIRI at 0.8174 (95% CI: 0.73–0.90), PIV at 0.83 (95% CI: 0.73–0.89), and SII at 0.74 (95% CI: 0.64–0.85) (Figure 2). Optimal cutoff values, determined using the maximum Youden index, were 5815 for the GINI, 1038 for the SII, 1624 for the SIRI, and 625 for the PIV.

Clinicopathological features and laboratory parameters, including prognostic indices, were compared between the low- and high-GINI cohorts (Table 1). The GINI was found to be low in 62.1% of the patients (<5815). High GINI (≥5815) was more prevalent among elderly patients, those with poor ECOG PS (≥2), those with multifocal tumor presence, individuals who underwent gross total resection, received adjuvant chemotherapy and radiotherapy, who had wild-type IDH status, and exhibited elevated SIRI, SII, and PIV values, demonstrating significant clinical associations (*p* < 0.005). Strong correlations were identified between the GINI and SII (r = 0.694, *p* < 0.001), SIRI (r = 0.516, *p* < 0.001), and PIV (r = 0.657, *p* < 0.001).

### 3.2. Survival Analysis

**Figure 3:**
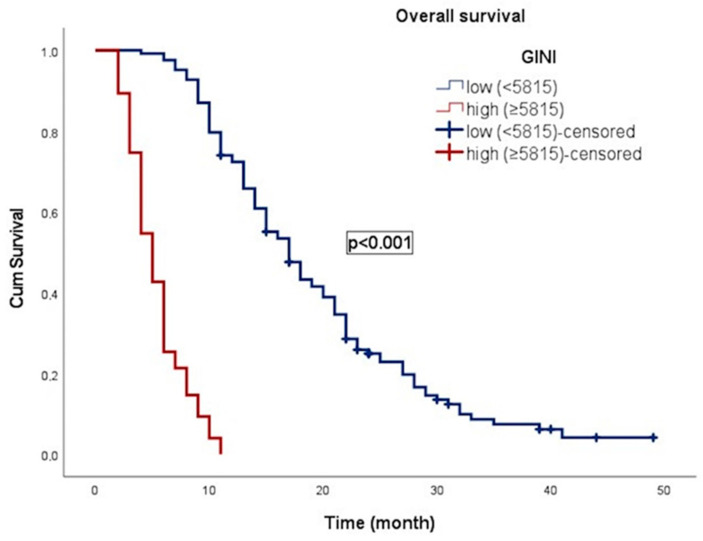
Kaplan–Meier curve illustrating the overall survival of patients classified according to GINI.

**Figure 4:**
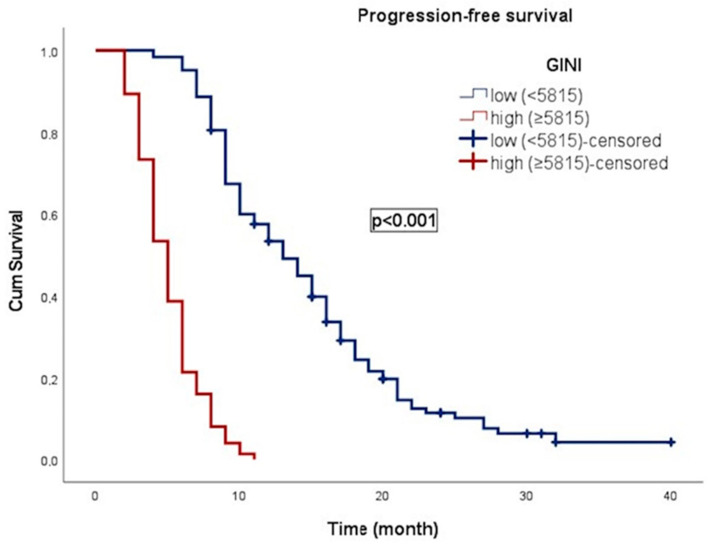
Kaplan–Meier curve illustrating the progression-free survival of patients classified according to GINI.

In an average follow-up duration of 13.7 months (95% CI: 2.1–49.0), progression occurred in 117 patients (59.1%), and 183 patients (92.4%) died. In patients with gr4 adult-type diffuse glioma, the median OS was 11.0 ± 0.8 (9.4–12.6) months. OS was 17.0 ± 1.1 months (14.8–19.3) in patients with low GINI and 5.0 ± 0.4 months (4.3–5.7) in patients with high GINI. Patients with low GINI exhibited significantly longer OS than those with high GINI (*p* < 0.001). In addition, the median PFS was 9.0 ± 0.4 (8.2–9.8) months. PFS was 13.0 ± 1.1 (10.9–15.1) months in patients with low GINI and 5.0 ± 0.3 (4.4–5.6) months in patients with high GINI. Patients with low GINI scores exhibited significantly longer PFS than those with high GINI scores (*p* < 0.001). The Kaplan–Meier survival curves for OS and PFS stratified by low and high GINI groups are shown in Figure 3 and Figure 4, respectively.

The clinical and laboratory parameters affecting OS in patients with gr4 adult-type diffuse glioma were investigated using a univariate Cox proportional hazards model (Table 3). In univariate analysis, age, ECOG PS, tumor focality, administration of adjuvant chemotherapy and radiotherapy, type of surgical procedure, IDH mutation status, presence of ATRX loss, GINI, SII, SIRI, and PIV were found to be significantly associated with overall survival (*p* < 0.05). In multivariate analysis, age, administration of adjuvant therapy, IDH mutation status, GINI, and SIRI remained significantly associated with overall survival (*p* < 0.05).

In the univariate Cox proportional hazards model, the following factors were significantly associated with PFS (*p* < 0.05): age, ECOG PS, tumor focality, adjuvant chemotherapy and radiotherapy, surgical procedure, IDH mutation status, ATRX loss, GINI, SII, SIRI, and PIV. In multivariate analysis, significant associations with PFS (*p* < 0.05) were found for age, adjuvant chemotherapy and radiotherapy, surgical procedure, IDH mutation status, and the GINI (Table 4).

Both univariate and multivariate analyses revealed that high GINI and high SIRI were adverse prognostic factors associated with reduced OS. Compared with other prognostic indices, an elevated GINI value independently constitutes a risk factor for both PFS and OS in patients with gr4 adult-type diffuse glioma. Furthermore, it is a robust predictor of adverse clinical outcomes.

## 4. Discussion

Gr4 adult-type diffuse glioma, including IDH-mutant gr4 astrocytoma and IDH-wt GBM gr4, represents a significant entity within CNS tumors and remains a highly fatal disease with a median survival expectancy of only 9–15 months despite ongoing clinical research endeavors [2,3]. Its aggressive nature and intrinsic resistance characteristics frequently result in disease progression, even after surgical resection of the primary tumor, accompanied by radiotherapy and adjuvant temozolomide therapy [4]. Recurrence is common, and patients often exhibit partial response to advanced therapies during subsequent monitoring [5,6,7]. Prognostic factors, including age, tumor size, feasibility of achieving complete tumor removal, O6-methylguanine-DNA methyltransferase (MGMT) methylation status, and particularly IDH mutation (more prevalent in secondary glioblastomas), markedly influence patient prognosis [5]. Nevertheless, the identification of markers capable of prognostically stratifying patients, anticipating clinical outcomes and survival in advance, and guiding clinicians in selecting optimal treatment strategies remain critically imperative.

Examining the evolution of cancer and prognostic biomarker research over the past decade, the field initially designed marker combinations based on immune-inflammation, and focused on cancer prognosis. This phase was followed by the development of indices that utilized multiple parameters. Subsequently, hypotheses were formulated by incorporating markers that reflect nutritional status into these combinations.

In a meta-analysis by Guo et al. on patients with GBM, high pretreatment NLR values were found to be associated with decreased survival [16]. Similarly, a retrospective observational study by Duan et al. on 281 patients with GBM demonstrated the prognostic capability of the NLR on clinical outcomes [20]. These findings were further supported by a meta-analysis conducted by Jarmuzek et al. [21] and an observational study by Yang et al. on low- and high-grade gliomas [23]. Furthermore, Bispo et al., in their systematic review, showed the detrimental impact of high PLR values on survival in GBM [17]. Combined indices such as the AGR and CAR, which emphasize combinations of markers that directly reflect the cancer–nutrition relationship, including CRP, albumin, and globulin, have also been investigated in GBM. A retrospective observational study by Yalikun et al. involving 126 GBM patients supported the notion that decreased albumin levels and AGR are independent predictors of poor survival outcomes [26]. Similarly, a large cohort meta-analysis by Liu et al. demonstrated that lower albumin and AGR levels were associated with decreased survival [27]. Additionally, a study by Liao et al. on colorectal cancer indicated that elevated CAR correlates with worse PFS and OS outcomes [28]. Overall, these studies aimed to elucidate cancer biology and its clinical implications using indices designed based on immune-inflammatory or nutrition-based hypotheses. This methodology has yielded precise scientific discoveries that offer prognostic and predictive insights across a diverse array of cancer types.

Recently, studies focusing on the prognostic potential of indices such as the SII, SIRI, and PIV in GBM research have been published. These indices are formed by combining three or more markers and have shown promise as prognostic biomarkers in various GBM studies [18,19,20,24]. In a study conducted by Shi et al. involving 232 GBM patients [18], similar to the study by Duan et al. [20], it was suggested that an elevated SII correlated with reduced survival rates and demonstrated superior predictive capabilities compared to binary indices, such as the NLR, PLR, and MLR. Subsequently, Yang et al. demonstrated in a follow-up study that the SII might possess an enhanced predictive capacity for survival in GBM compared to indices such as NLR and PLR [24]. In a recent study by Wang et al. involving 291 GBM patients who underwent gross total resection and utilizing propensity score matching analysis, it was observed that elevated preoperative SIRI values were significantly associated with reduced survival rates, surpassing the predictive ability of the NLR [22]. The findings from the study conducted by Topkan et al. on 204 patients with GBM substantiated the notion that higher values of the PIV, which integrates four markers, robustly predicted adverse clinical outcomes [25]. Subsequent to these studies, the concept arose that newly developed indices, such as the SII, SIRI, and PIV, demonstrated superior prognostic accuracy and predictive capabilities compared to binary indices. This improvement may be attributed to their formulation being based on a more integrated formula that is less susceptible to immediate fluctuations.

Insights from current studies have highlighted the potential for creating functional and multidimensional prognostic predictive indices through comprehensive evaluation of markers that can interact indirectly, akin to pieces of a puzzle, within the different physiological and biological conditions of each patient. As demonstrated in numerous studies, nutrition plays a role as significant as that of the immune system and inflammation processes in the clinical outcomes of cancer treatment. Comprehensive indices that incorporate immune, inflammatory, and nutritional parameters can be considered to provide accurate and reliable predictions. In this context, introducing a different perspective in cancer management, the GINI, designed for the first time by Topkan et al. [29], has garnered attention for its superior predictive capabilities in determining survival in a study involving patients with NSCLC. The results revealed that patients with high GINI had significantly shorter median PFS and OS than those with low GINI, indicating the potential of the GINI as an independent predictor of clinical outcomes in patients with stage IIIB/C NSCLC [29]. The current study has the potential to offer valuable and improvable contributions to future oncological research designs, as it represents the first adaptation of the GINI to the field of central nervous system tumors as a novel prognostic index.

The findings of this study support the notion that high GINI values in patients with gr4 adult-type diffuse gliomas are strongly associated with worse PFS and OS outcomes. The higher prevalence of negative risk factors, such as advanced age, low ECOG PS, and multifocal tumors that complicate maximal surgical resection, in patients with elevated GINI values for grade 4 adult-type glial tumors serves as a compelling illustration of the prognostic capability of the GINI. Univariate and multivariate analyses revealed that the GINI has a stronger impact on clinical outcomes than combined immuno-inflammatory markers, such as the SII, SIRI, and PIV. This effect can be explained by the presence of markers, such as CRP and albumin, which directly symbolize the connection between nutritional status and cancer cachexia, thereby enhancing the predictive potential of the GINI. The simplicity and availability of calculating the GINI using routine blood tests make it an appealing candidate for integration into clinical practice. This capability could potentially provide clinicians with a valuable tool for stratifying risk and selecting treatments for patients diagnosed with IDH-mutant gr4 astrocytoma and IDH-wt GBM gr4. Consequently, the GINI distinctly underscores the prognostic implications and significance of nutritional status in cancer dynamics from a novel perspective, thereby highlighting avenues for future research.

The capacity and adequacy of this study are highlighted by consistent and positive aspects, such as a significant number of cases in a relatively rare tumor group, adherence of all patients to international standard treatment protocols in surgical and medical approaches, radiological and clinical evaluations conducted by a tertiary cancer center with a multidisciplinary active tumor board, and consultation of supportive–nutritional treatments by an experienced team in the later stages of the disease. However, the current study had certain limitations. Its retrospective design and single-center nature may affect the balanced distribution of cases, application of more detailed statistical analyses, and generalizability of the results. Owing to cost-effectiveness and technical constraints, tumor-related biomarkers, such as MGMT, which could directly impact prognosis, were not included in the study, potentially weakening its robustness. This index, which is based on a multivariate formula, includes markers that may indirectly influence one another. Moreover, some of these markers can activate intrinsic chemokines or cytokines in the body, thereby affecting immune responses and the clinical course of cancer via different mechanisms. The lack of internationally established standard cutoff values for each marker can also be considered a limitation. Furthermore, certain issues may have been overlooked, such as mild infections without clinical symptoms at the time of parameter measurements, individual variations in immune system changes, variable momentary fluctuations in marker levels, and the absence of an internal validation group. The possibility of bias in the GINI groups owing to differences in advanced-line treatment options should also be considered. Specifically, several factors complicate the interpretation of clinical outcomes, including tolerance issues related to the continuation of advanced-line treatments in patients who experience progression, challenges related to the feasibility of re-surgery or re-radiotherapy, and the limited availability of second-line treatment options. In the future, designing studies based on larger cohorts that include an internal validation group could yield more accurate and convincing insights into the prognostic significance and predictive capacity of the GINI.

## 5. Conclusions

This study has provided evidence to support the robust predictive capabilities and dependable prognostic outcomes of the GINI model, which integrates intricate elements of immune-inflammation and nutrition, in predicting the survival of patients with IDH-mutant gr4 astrocytoma and IDH-wt GBM gr4. Both the cancer itself and the side effects of cancer treatment, such as cachexia and nutritional deficiencies, can adversely affect cancer prognosis. The GINI has once again highlighted that nutritional parameters, when used in conjunction with immune-inflammation markers, can provide reliable and consistent contributions to understanding the clinical course of cancer. Improving the quality of life and treatment outcomes of cancer patients hinges critically on maintaining nutritional balance and bolstering the immune system. With its noninvasive, easily accessible, cost-effective, and repeatable characteristics, the GINI can provide valuable information that guides clinicians in gr4 adult-type diffuse glioma management.

## Figures and Tables

**Table 1:** Comparison of sociodemographic and clinicopathological characteristics of patients classified according to the GINI (all patients, n = 198).

Variables			GINI		
			Low (<5815)	High (≥5815)	*p*
Age (year), n (%)	<60	98 (49.5%)	69 (56.1%)	29 (38.7%)	0.013
	≥60	100 (50.5%)	54 (43.9%)	46 (61.3%)	
Sex, n (%)	Male	114 (57.6%)	72 (58.5%)	42 (56.0%)	0.419
	Female	84 (42.4%)	51 (41.5%)	33 (44.0%)	
Comorbidity, n (%)	No	121 (61.1%)	80 (65.0%)	41 (54.7%)	0.097
	Yes	77 (38.9%)	43 (35.0%)	34 (45.3%)	
Smoking status, n (%)	No	131 (66.2%)	85 (69.1%)	46 (61.3%)	0.167
	Yes	67 (33.8%)	38 (30.9%)	29 (38.7%)	
ECOG PS, n (%)	0–1	156 (78.8%)	110 (89.4%)	29 (38.7%)	<0.001
	2	42 (21.2%)	13 (10.6%)	46 (61.3%)	
Laterality, n (%)	Left	106 (53.5%)	69 (56.1%)	37 (49.3%)	0.218
	Right	92 (46.5%)	54 (43.9%)	38 (50.7%)	
Localization, n (%)	Temporal	45 (22.7%)	28 (22.8%)	17 (22.6%)	0.884
	Frontal	56 (28.3%)	36 (29.3%)	20 (26.7%)	
	Parietal	76 (38.4%)	47 (38.2%)	29 (38.7%)	
	Occipital	21 (10.6%)	12 (9.8%)	9 (12.0%)	
Tumor focality, n (%)	Unifocal	153 (77.3%)	101 (82.1%)	23 (30.7%)	0.029
	Multifocal	45 (22.7%)	22 (17.9%)	52 (69.3%)	
The type of surgery, n (%)	Subtotal	101 (51%)	70 (56.9%)	31 (41.3%)	0.003
	Gross total	97 (49%)	53 (43.1%)	44 (58.7%)	
Adjuvant radiotherapy, n (%)	No	20 (10.1%)	1 (0.8%)	19 (25.3%)	<0.001
	Yes	178 (89.9%)	122 (99.2%)	56 (74.7%)	
Adjuvant chemotherapy, n (%)	No	39 (19.7%)	3 (2.4%)	36 (48.0%)	<0.001
	Yes	159 (80.3%)	120 (97.6%)	39 (52.0%)	
IDH mutation, n (%)	Mutant	42 (21.2%)	41 (33.3%)	1 (1.3%)	<0.001
	Wild-type	156 (78.8%)	82 (66.7%)	74 (98.7%)	
ATRX loss, n (%)	No	134 (67.7%)	79 (64.2%)	55 (73.3%)	0.120
	Yes	64 (32.3%)	44 (35.8%)	20 (26.7%)	
SII, n (%)	<1038	118 (59.6%)	106 (86.2%)	12 (16.0%)	<0.001
	≥1038	80 (40.4%)	17 (13.8%)	63 (84.0%)	
SIRI, n (%)	<1624	77 (38.9%)	72 (58.5%)	5 (6.7%)	<0.001
	≥1624	121 (61.1%)	51 (41.5%)	70 (93.3%)	
PIV, n (%)	<625	114 (57.6%)	102 (82.9%)	12 (16.0%)	<0.001
	≥625	84 (42.4%)	21 (17.1%)	63 (84.0%)	

Abbreviations: ECOG PS, Eastern Cooperative Oncology Group Performance Status; IDH, isocitrate dehydrogenase; ATRX, alpha-thalassemia/mental retardation syndrome X-linked; GINI, global immune-nutrition-inflammation index; SII, systemic immune-inflammation index; SIRI, systemic inflammation response index; PIV, pan-immune-inflammation value.

**Table 2:** AUC values for each index compared using ROC curve analysis.

	AUC	Std. Error	95% CI	*p*	Sensitivity (%)	Specificity (%)
GINI	0.861	0.044	0.776–0.947	<0.001	100.0	62.1
SII	0.738	0.055	0.636–0.846	0.002	87.5	62.5
SIRI	0.812	0.042	0.726–0.895	<0.001	100.0	57.4
PIV	0.803	0.042	0.726–0.886	<0.001	87.5	59.5

Abbreviations: AUC, area under the curve; Std., standard deviation; CI, confidence interval.

**Table 3:** Cox regression model of OS in patients with grade 4 adult-type diffuse glioma.

	Overall Survival							
	Univariate				Multivariate			
	HR (95% CI for HR)			*p*	HR (95% CI for HR)			*p*
Age	0.534	0.396	0.719	<0.001	0.583	0.415	0.820	0.002
Sex	0.997	0.743	1.338	0.983	-	-	-	-
Comorbidity	1.227	0.912	1.653	0.177	-	-	-	-
Smoking status	1.119	0.824	1.518	0.472	-	-	-	-
ECOG PS	2.585	1.799	3.716	<0.001	0.759	0.506	1.140	0.184
Laterality	1.040	0.777	1.392	0.793	-	-	-	-
Localization	1.060	0.904	1.242	0.476	-	-	-	-
Tumor focalit	1.649	1.173	2.319	0.004	1.387	0.967	1.990	0.075
The type of surgery	0.552	0.411	0.742	<0.001	0.770	0.549	1.080	0.130
ATRX loss	0.614	0.446	0.846	0.003	1.093	0.753	1.587	0.639
IDH mutation	0.193	0.123	0.301	<0.001	0.313	0.183	0.534	<0.001
Adjuvant radioterapy	0.132	0.079	0.221	<0.001	0.353	0.202	0.618	<0.001
Adjuvant chemotherapy	0.124	0.083	0.185	<0.001	0.448	0.269	0.746	0.002
GINI	14.110	8.963	22.213	<0.001	8.132	4.690	14.100	<0.001
SII	4.404	3.197	6.067	<0.001	1.184	0.721	1.943	0.504
SIRI	3.501	2.527	4.850	<0.001	1.668	1.060	2.627	0.027
PIV	4.532	3.301	6.222	<0.001	1.258	0.741	2.133	0.396

Abbreviations: ECOG PS, Eastern Cooperative Oncology Group Performance Status; IDH, isocitrate dehydrogenase; ATRX, alpha-thalassemia/mental retardation syndrome X-linked; GINI, global immune-nutrition-inflammation index; SII, systemic immune-inflammation index; SIRI, systemic inflammation response index; PIV, pan-immune-inflammation value.

**Table 4:** Cox regression model of PFS in patients with grade 4 adult-type diffuse glioma.

	Progression-Free Survival							
	Univariate				Multivariate			
	HR (95% CI for HR)			*p*	HR (95% CI for HR)			*p*
Age	0.578	0.429	0.778	<0.001	1.527	1.088	2.143	0.014
Sex	1.038	0.773	1.394	0.802	-	-	-	-
Comorbidity	1.252	0.929	1.688	0.140	-	-	-	-
Smoking status	0.977	0.719	1.329	0.884	-	-	-	-
ECOG PS	2.615	1.823	3.751	<0.001	0.769	0.506	1.170	0.220
Laterality	1.006	0.752	1.347	0.968	-	-	-	-
Localization	1.006	0.858	1.180	0.939	-	-	-	-
Tumor focality	1.438	1.023	2.022	0.036	1.272	0.885	1.829	0.193
The type of surgery	0.454	0.333	0.618	<0.001	0.663	0.471	0.934	0.019
ATRX loss	0.602	0.435	0.833	0.002	1.088	0.747	1.585	0.659
IDH mutation	0.305	0.205	0.456	<0.001	0.517	0.320	0.835	0.007
Adjuvant radiotherapy	0.152	0.092	0.252	<0.001	0.372	0.214	0.647	<0.001
Adjuvant chemotherapy	0.157	0.107	0.232	<0.001	0.449	0.265	0.764	0.003
GINI	9.110	6.152	13.492	<0.001	5.827	3.524	9.633	<0.001
SII	3.866	2.816	5.307	<0.001	0.969	0.566	1.658	0.908
SIRI	2.877	2.095	3.953	<0.001	1.465	0.950	2.259	0.084
PIV	4.074	2.971	5.587	<0.001	1.204	0.683	2.123	0.521

Abbreviations: ECOG PS, Eastern Cooperative Oncology Group Performance Status; IDH, isocitrate dehydrogenase; ATRX, alpha-thalassemia/mental retardation syndrome X-linked; GINI, global immune-nutrition-inflammation index; SII, systemic immune-inflammation index; SIRI, systemic inflammation response index; PIV, pan-immune-inflammation value.

## Data Availability

The datasets used in this study can be made available by the corresponding author upon reasonable request, with permission from the Clinical Oncology Department of HSUAERH.
